# Temporal order judgments and presaccadic shifts of attention: What can prior entry teach us about the premotor theory?

**DOI:** 10.1167/jov.22.12.6

**Published:** 2022-11-03

**Authors:** Paul Fisher, Thomas Schenk

**Affiliations:** 1 Lehrstuhl für Klinische Neuropsychologie, Ludwig Maximilians Universität, Munich, Germany

**Keywords:** attention, saccades, premotor, prior entry, temporal order judgments, action attentional link, presaccadic, cueing

## Abstract

A temporal order judgment (TOJ) 2-alternative forced choice design was used to examine presaccadic shifts of attention. Prior work on the premotor theory of attention (PTA) has predominantly focused on single-target discrimination tasks as a tool to measure accuracy and shifts of attention. It is important to demonstrate that the PTA is effective across attentional tasks that have been shown to be reliable in other contexts. Therefore, it was decided to use a perceptual task that probes multiple locations simultaneously and can equally be used to examine spatial spread of attention in more detail. In typical TOJ studies, prior entry is the metric used to measure an attentional effect. Prior entry is the biasing of temporal perception toward an attentionally cued location. This generally manifests as observers processing events at the cued location more rapidly, altering their perspective of temporal order. Participants were required to prepare saccades toward one of four targets, two of which would light up either synchronously or sequentially after a GO signal but before saccadic execution. Results demonstrated that in conditions with critical stimulus onset asynchronies, saccade preparation had a significant effect on performance. Prior entry effects were observed at saccade congruent locations with probes at these locations being typically perceived earlier than probes presented at a neutral location. These effects were not observed in control trials without a saccade. A further spatial effect was demonstrated for the attentional modulation, suggesting that this effect is restricted predominantly to horizontal configurations. Overall, results demonstrated that presaccadic attention is effective at eliciting a prior entry effect in TOJ designs and that such effects are more pronounced when the probes are distributed across the two lateral hemifields.

## Introduction

We live in a stimulus-rich and dynamic world that requires constant shifts of our attention to navigate and prioritize events across our environment. Consequently, our attention system operates via a selection mechanism that can rapidly process locations and objects in real time. Eye movements are one such process by which selection of desired or highly salient events can be facilitated. Typically, this is accomplished by goal-directed saccades in order to foveate a location and thereby increase the level of fidelity for the perceptual representation of the stimulus at the foveated location (Bisley, 2011; Kowler, Anderson, Dosher, & Blaser, 1995). The exact mechanisms that contribute to this relationship between motor systems and attention have received widespread interest, with an extensive library of research demonstrating a strict coupling between visual attention and motor actions, known as the premotor theory of attention (PTA; Craighero & Rizzolatti, 2005; Craighero, Fadiga, Rizzolatti, & Umiltà, 1999; Rizzolatti, Riggio, Dascola, & Umiltá, 1987).

One claim of the PTA contends that there is an intrinsic link between attention networks and the oculomotor system to the degree that the preparation of motor actions is both sufficient and necessary to result in a shift of attention (for a review, see Smith & Schenk, 2012). This mandatory coupling predicts that the preparation of a motor action is functionally equivalent to a shift of attention, meaning that attentional resources will always coincide with the intended goal location of a movement, even prior to the movement's execution. Research has suggested that there is increased discrimination accuracy, an oft-used criterion to measure attention, at the endpoint of prepared motor actions. Indeed, an impressive array of studies demonstrated this phenomenon for both saccades and pointing movements (Baldauf & Deubel, 2008; Deubel, 2008; Hoffman & Subramaniam, 1995; Rolfs, Jonikaitis, Deubel, & Cavanagh, 2011; Rorden, Greene, Sasine, & Baylis, 2002; Shepherd, Findlay, & Hockey, 1986).

However, in most studies examining the PTA and visual attention, the measure for attention is the same—namely, a single target probe perceptual discrimination task. Restricting the research domain to a specific attentional measure has inherent drawbacks from a practical and a theoretical perspective. First, the claim of PTA and related hypotheses (e.g., Deubel & Schneider, 1996) is that motor preparation is linked to a shift of attention to the goal of the action. Attention is a concept that transcends any specific perceptual tasks. Attention reflects a change, typically an improvement, in neural processing of specific information that should lead, in the domain of perception, to better and faster perceptual decisions. Importantly, these improvements are expected to be found in most if not all perceptual tasks. Nonetheless, the fact that performance in single-target probe discrimination tasks is improved at the intended endpoint of an action is certainly compatible with the presumed link between motor programming and attention. However, this claim would be more compelling if it could be shown that such improvements are not restricted to a few, highly similar tasks. Instead, one would like to see evidence that the improvements also extend to quite different tasks for which attentional modulation has been demonstrated before in other contexts. Thus, in order to further substantiate the theory of the PTA, as well as related hypotheses claiming a direct link between action and attention (hereafter referred to as the action attentional link, or AAL), it would be advantageous to examine other perceptual tasks, such as those requiring multiple discrimination targets and observe any subsequent behavioral impacts.

The present study aims to explore attentional effects that are attributed to the PTA using a method that probes the level of attention at two distinct locations simultaneously. It was therefore decided to measure temporal order judgments (TOJs) in order to examine attentional effects that result from cueing one of two synchronous or sequential probes. The effect of such attentional cueing on the perceived temporal order of a sequence of events is known as the *prior entry* effect (Schneider & Bavelier, 2003; Spence & Parise, 2010). Prior entry is a phenomenon first coined by Titchener (1908) that describes how a shift in attention can alter the perception of temporal order, even when stimulus onset is objectively synchronous. In practice, participants cannot judge with perfect fidelity the precise timing offset between two probes, but they can judge with some confidence which of two stimuli appeared first. The most common TOJ tasks simply ask participants to indicate which of two sequentially presented stimuli appears first (see Spence & Parise, 2010, for a review of the paradigm). Alternatively, the examiner can also instruct observers to indicate the stimuli that seemed to appear last. As will be shown below, there are advantages to using both types of instructions. Research has found that, with both endogenous and exogenous cues, participants are more likely to indicate that the attended stimulus appeared first (Shore et al., 2001; Zackon, Casson, Zafar, Stelmach, & Racette, 1999) even when the two stimuli were presented simultaneously. Indeed, robust effects have been demonstrated for a variety of sensory modalities (Alais & Cass, 2010; Redden, d'Entremont, & Klein, 2017; Spence, Shore, & Klein, 2001; Zampini, Shore, & Spence, 2005; Zampini et al., 2007). The degree to which participants favor their perception over the objective temporal difference is called the prior entry bias.

Common AAL designs, such as the experiment by Deubel and Schneider (2003), require participants to perform a saccade or pointing movement to one of multiple target placeholders and instruct them to make a perceptual decision related to an attribute of a single probe (e.g., deciding between two possible symbols, E or Ǝ, or between tilted gabor patches). In addition to providing a novel alternative to the standard AAL discrimination tasks mentioned above, a TOJ design allows attention to be probed in multiple locations simultaneously. One benefit of this design is that, if successful, it demonstrates that PTA effects are not limited to single-target probe discrimination tasks. Moreover, gathering information about how participants perceive the difference between two probes gives valuable information about the spatial spread of attention. This means that within a single trial, the spatial bias can be more accurately ascertained. For example, research has demonstrated that spatial bias can be measured with TOJ tasks for visuospatial neglect patients as a method to measure lesion deficits in the visual field (Van der Stigchel & Nijboer, 2018). Therefore, exploiting the prior entry phenomenon may afford an opportunity to examine the spatial distribution of the attentional focus induced by action by explicitly demanding a discrimination of both a cued and an uncued location. By virtue of using a secondary, equally relevant probe, participants are discouraged from deliberately shifting their attention to the cued location. However, PTA assumes that the link between the endpoint of a saccade and the position of maximum attention is mandatory, is automatic, and does not require deliberate spatial shifts of attention. Thus, PTA predicts that in a TOJ paradigm, the probe that is aligned with the endpoint of the saccade will be typically perceived as having appeared before the other probe even if the two probes were presented at roughly the same time. Confirming such a prior-entry effect in a PTA paradigm would provide compelling evidence that the attentional boost at the saccade endpoint is indeed automatic and independent of cue-guided, deliberate shifts of attention.

In our study, participants performed a TOJ task that required preparatory saccades in a similar manner to classic AAL experiments. In line with the PTA, we arrived at the following predictions. First, following the principles of prior entry, we would predict that participants would have an increased TOJ accuracy when a saccade was prepared toward the location of the first-to-appear stimulus. Second, when a saccade was prepared toward the second-to-appear stimulus, we predict that there would be interference between the prior entry effect and the objective sequence of events, culminating in worse performance at this location. Third, we would predict that no attentional benefit would be found when a saccade was directed toward a separate, neutral location. Importantly, if successful, this design will demonstrate that the link between motor action preparation and attention is not reliant on perceptual single-target discrimination tasks. Furthermore, it provides a paradigm to compare directly, within one trial, the distribution of attention between two different positions. Attentional effects are always relative effects. We infer that attention has been allocated to position A but not B by observing that performance for the same task is better at A than performance at B. Only a *difference* in performance provides evidence for attention. Thus, it is inherent in the concept of spatial attention that we wish to compare performance for targets presented at different locations. The TOJ task in conjunction with a PTA paradigm allows us to compute this difference within a single trial.

Additionally, in some PTA studies, we suspect that attention is allocated to more than one location and we would like to compare the attentional boost provided to those different locations. One example of such an attentional multifocus PTA study was recently provided by Hanning et al. (2018). In this study, the authors wanted to explore how the attentional effects of moving two different effectors (i.e., the eye and the hand) would interact. When using a one-probe discrimination paradigm, such comparisons between locations must be inferred by comparing performance averages computed across many trials that have been binned with respect to the target location used in each respective trial. In such a paradigm, trial-by-trial fluctuations contaminate the location-by-location comparison. If successful, the paradigm examined here can allow a direct comparison of attentional effects at different positions within one trial.

## Methods

### Participants

Ten healthy adults (age 18–35, 5 females), including one of the researchers, with normal or corrected-to-normal vision were recruited from the LMU Munich via word of mouth and email correspondence. Participants were compensated for their time (€10 per hour or student credits), and written informed consent was obtained in accordance with the Declaration of Helsinki. Participants took part in all three sessions, and thus, subsequent analysis was based on paired-samples statistics.

### Apparatus

A high-resolution 24.5-in. (63.5-cm) Dell Alienware gaming monitor (model AW2518H) with a 240 Hz refresh rate was used to ensure the highest temporal resolution of visual presentation possible (Poth et al., 2018). This was complemented by a Quadro p4000 GPU with compatible Nvidia GeForce G-Sync software. The experiment was designed using MATLAB 2015b and written using Psychophysics Toolbox (PTB) extensions (Brainard, 1997; Kleiner, Brainard, & Pelli, 2007). Using specialized functions, PTB allows users to bypass restrictions on a monitor refresh rate by using variable refresh rate (VRR) support, also known as adaptive sync mode. Typically, an experiment is restricted in timing by the refresh rate of the monitor (e.g., a 60 Hz monitor will show a stimulus for a minimum of 16.666 ms). The combination of a monitor with high temporal resolution, G-Sync, and VRR support enables an experiment to change the visual display on a monitor very rapidly. In brief, this allowed for a screen-flip rate and therefore a minimum presentation time to be accurate to around ∼1 ms. The only bottleneck to this system is that a frame must be longer than the minimum of one frame for the monitor refresh rate. In our setup, this was 240 Hz, meaning that requested frames below ∼4 ms would not be possible but any interval of ∼1 ms above this was theoretically obtainable. See supplementary materials for further details.

An iView *REDn Scientific* eyetracking system developed by SensoMotoric Instruments (SMI; Niehorster & Nyström, 2020) was used for collecting eye movement data on saccades and fixation. The eyetracker is attached to the bottom of the monitor, which is ∼58 cm from the participants and requires a USB 3.0 port. Participants were also required to use a chinrest to avoid changes in head position during the experiment. The experiments were conducted, without distraction, in a dark lab under the experimenter's supervision. Furthermore, the optimal conditions recommended by the *REDn Scientific* system user guide were followed to improve eyetracking operating precision.

### Stimuli

**Figure 1.**
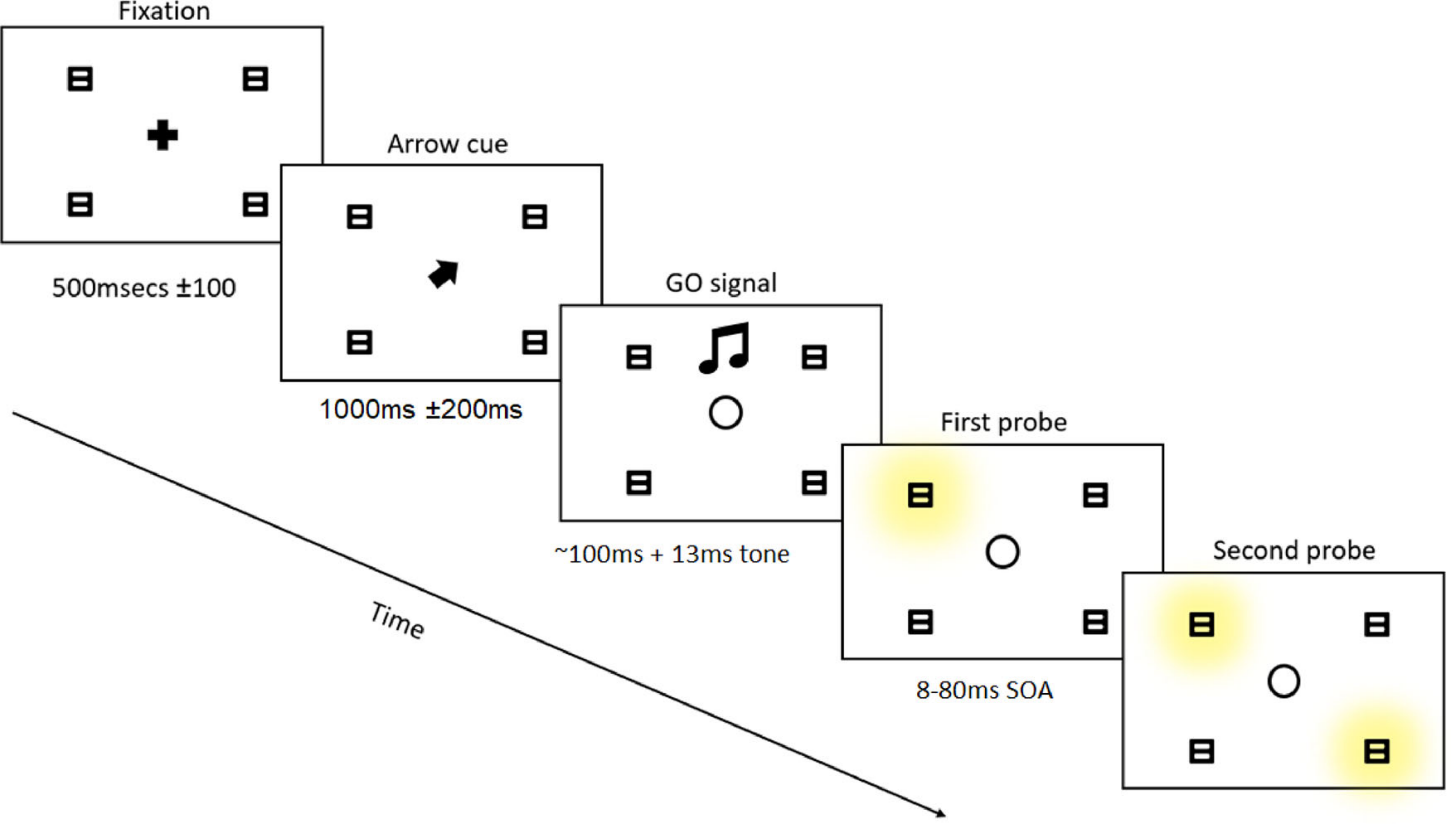
This schematic illustrates a typical trial type that was used for all three sessions. Trials would begin with a fixation cross that would be displayed for a minimum of 500 ± 200 ms (it required participants to fixate for 500 ms without a break in order to continue). Following this, on cued trials, participants would then see an arrow that would point to one of the four target locations (1,000 ± 200 ms). (1) For Session 1, participants would receive no GO signal and the display would switch directly to the first probe display. (2) For Session 2, participants would hear a short (100-ms) tone that had a ∼13-ms jitter. (3) For Session 3, the display was identical to Session 2. Finally, in all three sessions, participants would then be shown the two target probes. They could be presented horizontally, vertically, or diagonally (8–80 ms).

Each trial began with a fixation cross (0.67° × 0.67°) presented in white (RGB = 255,255,255) on a black (RGB = 0,0,0) background. There were four gray (RGB = 63,63,63) placeholder target “8” symbols (1.2° × 1.2°) that framed the display forming a square (see Figure 1). The target “8” symbols, during trial conditions, could also change to white (RGB = 255,255,255) for a short period of time. The arrow cue was a white chevron V-shape and appeared centrally (0.67° × 0.67°). The arrow cue was additionally mipmap bilinear filtered in order to reduce pixelation in MATLAB. The GO signal was a short tone at a frequency of 500 Hz across two channels that played for 100 ms from a low-latency high-precision sound driver in PTB.

### Design and procedure

A within-subject design was implemented with testing completed in one sitting (∼2.5 hr), which required participants to complete three sessions. The three sessions (described in detail below) were designed to examine the participants’ ability to perform TOJ tasks while we attempted to manipulate their attentional focus. The first session was primarily used to determine for each participant the minimally required temporal asynchrony necessary to produce above-chance accuracy in their TOJ. The findings from Session 1 allowed us to adjust the temporal parameters of subsequent experiments to the individual skills of our participants and thereby ensure that we created optimal conditions to measure potential modulatory effects of our experimental (i.e., attentional) manipulations. This was achieved using the method of constant stimuli, with SOAs ranging between 8 and 80 ms being tested in a random order to determine a perception threshold. Session 1 also provided an opportunity to examine the influence of an irrelevant arrow cue on target discrimination. This adaptive procedure meant that participants did not need to be highly trained as their individual performance was integrated into subsequent testing. The goal of the second session was to examine the potential influence of eye movements on TOJs. To do this, we combined the task from Session 1 with a saccade task. In this session, we compared three conditions. First, the *simultaneous* condition: Here the question was whether an attentional boost created by the saccade might in fact produce a temporal bias in a judgment of events that objectively occurred at the same time. The second condition was called the *JND* condition (see Session 2). In this condition, we chose time intervals between the two events that were at the TOJ threshold for each of our participants. Accordingly, the intervals chosen differed from participant to participant. The last condition (*ceiling* condition) employed the time interval at which all participants could easily and correctly judge which of the events occurred first (80 ms). Our third session served as a control condition for Session 2. The third session was identical in almost all respects to Session 2 but did not include a saccade task.

#### Session 1

The purpose of Session 1 was to find the best (e.g., most sensitive) condition under which an attentional effect on TOJ could be measured when SOA was sequential rather than synchronous. To this end, we determined for each observer the temporal interval at which this observer could reliably (i.e., with a threshold of 75%) identify the stimulus that was presented first or alternatively the interval at which observers could reliably identify the stimulus that appeared second. Furthermore, it was deemed important to rule out attentional effects that might have been caused by an arrow cue alone. The session was therefore subdivided into two conditions (*cue* vs. *no* *cue*). The arrow cue was implemented here to examine the potential attentional impact of some of our experimental design choices (e.g., the use of an arrow to indicate the goal location for the instructed saccade in later experiments). Participants were given a practice task before beginning the experiment. Only after an acceptable threshold was reached, and the experimenter was satisfied that they understood the requirements, did the testing begin. In both the *cue* and *no-cue* conditions, 192 trials had to be carried out by all participants across three blocks each (counterbalanced for order) for a total of 384 trials. At the onset of each block, they were instructed as to whether it was a *cue* or *no-cue* condition. Both conditions required participants to maintain central fixation on the cross for 500 ms (jittered ± 100 ms), after which, the *cue* condition would display an arrow for 1,000 ms (jittered ± 200 ms) that would point with equal probability to any of the four placeholder “8”s. This was followed by two of the placeholder “8”s changing to white and remaining white until the decision had been made. This was to avoid any attentional interference that could result from target offset. A variable SOA of between 8 and 80 ms (1-ms increments) was used to separate the probe events onset. The *no-cue* condition would operate identically, but without the arrow cue. It was the job of the participant to make a temporal order judgment and indicate by button-press (see below) which of the two probe events appeared first or second, depending on the instructions of that block. Evidence has suggested that alternating between “*which came first?*” and “*which came second?*” trial types can help reduce response bias effects and minimize decision preferences on SOAs with high uncertainty (Spence & Parise, 2010). To avoid confusion across blocks, a label appeared at the top of each trial with either a “1” (*which came first?*) or “2” (*which came second?*) to indicate which trial type they were currently performing, and it was stressed during training to refer to this element in case of confusion over trial condition. It was also made clear to the participants on *cue* condition trials to ignore the arrow and to maintain fixation throughout the trial (measured with an eyetracker).

Responses were recorded using keyboard arrow keys. Importantly, all directions were used for this experiment. On trials where the two illuminated “8”s were on opposite sides of the visual vertical meridian, left and right arrows were required, and on trials where the target “8”s appeared on the same side of the vertical meridian, up and down arrows were required. Thus, there were three directional possibilities: horizontal (left and right), vertical (up and down), and diagonal (left and right). This was taught to the participants during training, and inappropriate keys were disabled during trials to help reiterate the concept and avoid extraneous errors (see supplementary material for a schematic of key mapping). All participants learned this response requirement without exception during the training phase. Finally, participants received feedback in the form of a green (correct) or red (incorrect) flicker to the fixation cross at the end of each trial. More details on how the data from Session 1 were employed to calculate the appropriate threshold value, determined for each observer and later used in the TOJ part of the following experiments, will be described in the section on data analysis.

#### Session 2

The second session was, for all intents and purposes, the most critical part of this study. The purpose of this session was to examine whether an attentional boost, via the preparation of a saccade, leads to a measurable attentional bias in a TOJ task. Furthermore, we hoped to exploit the spatially dispersed nature of the TOJ task to measure the spatial spread of any putative saccade-induced attentional boost relative to several targets as a function of the prior entry effect. The setup was functionally identical to Session 1 except for a few important differences. First, the participants were instructed that they would now be required to make saccades to the arrow-cued placeholder. A typical trial would be performed as follows: The participant maintains fixation (500 ms), followed by an arrow cue (1,000 ms, jittered ± 200 ms). Following the arrow cue preparation window, a tone would then be played (100 ms), which was an indication to perform the saccade as quickly and accurately as possible. The first placeholder “8” would illuminate after 85 ms (jittered ± 30 ms) from the onset of the tone and was quickly followed by the second placeholder “8” at one of three SOAs (also see Figure 1). The three SOAs were 0 ms (*simultaneous* condition), the interval determined for each individual separately with the JND staircase procedure from Session 1 (*JND* condition), and 80 ms (*ceiling* condition). The participant would then make the same TOJ as in the first task, although this time without feedback. The session was conducted for 12 blocks, with 72 trials per block for a total of 864 trials. Order of conditions was randomized within blocks, and the arrow cue pointed to the first target 25% of the time, to the second target 25% of the time, and randomly to the other two targets 50% of the time. Participants did not receive feedback on these trials. Once again, blocks were assigned to either a first or second TOJ judgment-response requirement (alternated block-by-block). The same response types (keyboard keys) were required as in Session 1 and a break was offered after six blocks had been completed.

#### Session 3

The final session was identical to Session 2, but without the requirement for participants to perform a saccade. It was conducted with the same timings as Session 2, and the only difference was the absence of the GO signal tone, and the arrow cue was displayed for a slightly shorter interval of 500 ± 100 ms. Instead, the participants maintained fixation throughout, verified via eyetracker. The purpose of this experiment was to examine the same SOA conditions used for Session 2 but without the saccade preparation. It therefore served as a direct no-saccade control condition. Additionally, there was no feedback given for Session 3, and the task of the participants remained the same. The Session 3 conditions will be referred to with the label No Saccade (NS) as *simultaneous NS*, *JND NS*, and *ceiling NS.* See Table 1.

**Table 1.** The conditions of the three experiments outlined for clarity. *Note*: Saccadic Stimulus refers to which of the probes the arrow points toward: the first-to-appear, the second-to-appear, or one of the two other irrelevant placeholders. Dimension is not included as a descriptor here but was required only for session 2. NS refers to “No Saccade.”

Experiment	Manipulation	Saccadic Stimulus		
		First	Second	Other
1	Cue	*Cue First*	*Cue Second*	*Cue Other*
	No cue	*n/a*	*n/a*	*n/a*
2	Simultaneous	*Simultaneous First*	*Simultaneous Second*	*Simultaneous Other*
	JND	*JND First*	*JND Second*	*JND Other*
	Ceiling	*Ceiling First*	*Ceiling Second*	*Ceiling Other*
3	Simultaneous—(NS)	*Simultaneous NS First*	*Simultaneous NS Second*	*Simultaneous NS Other*
	JND—(NS)	*JND NS First*	*JND Second*	*JND Other*
	Ceiling—(NS)	*Ceiling NS First*	*Ceiling NS Second*	*Ceiling NS Other*

### Eyetracking

All eyetracking data were examined offline after testing using SMI BeGaze v.3.4 proprietary software and MATLAB scripts. The iView *REDn Scientific* device records at 60 Hz and was used to ensure fixation was maintained by participants throughout the trials. Eyetracking data were of importance during Session 2, where it was used to determine that participants had made their saccade to the correct target and during the correct temporal window. The eyetracker was operated using its preprogramed functionality that allowed for tracking of both eyes without sacrificing fidelity. An optional feature was also selected that allowed for tracking to continue if one eye was closed, or briefly lost tracking. The eyetracker's gaze position accuracy is 0.4°. It measures position with a spatial resolution of 0.05°. Blinks were recorded and filtered.

Calibration was performed before all testing stages, and recalibration was required after breaks or if the participant removed their head from the chinrest. The calibration method used came as part of the SMI BeGaze v.3.4 proprietary software and displayed nine observation points. Participants were required to fixate a target observation point (a red circle), during which time the endpoint accuracy would be recorded by sampling visual angle coordinates of each eye for a total of 5 s. The fixation target would then move automatically onto the next calibration location. A mean fixation accuracy of less than 1° visual angle across all the observation points was the threshold for a successfully validated calibration.

The onset of a saccade was defined as the point in time when the movement of the eye exceeded both a velocity criterion (30°/s) and an acceleration criterion (8,000°/s). The offset of a saccade was defined as the point in time where the movement of the eye dropped below the defined velocity criterion. Therefore, saccade endpoints were calculated as the location at which a saccade offset was first recorded. The saccade endpoint needed to be recorded within a maximum of 1° visual angle around the target to be considered accurate. At the start of each trial, an online script would determine if participants were maintaining adequate fixation before continuing with the procedure. Therefore, trials would often have a fixation window longer than 500 ms if, for example, participants closed their eyes or rapidly blinked at the start of trials.

### Exclusion criteria

For Sessions 1 and 3, no saccades were required; therefore, trials were only excluded if a participant did not maintain a central gaze throughout the active trial. Trials that included eye movements pre- or posttrial, or during the decision phase, were not excluded as these were not considered part of the ongoing trial. For Session 2, data were excluded if one of the following errors occurred: endpoint outside placeholder, anticipation error, or other anomalous results. The saccade endpoint was required to land accurately within the threshold radius (1° visual angle) of the placeholder location. Anticipation errors were defined as abnormally early saccade onsets that were likely performed in error; this was calculated as the time from the GO signal to the time of the second target being illuminated + 40 ms. This also ensured that saccades appearing during the target illumination would be excluded. A trial was considered anomalous if the saccade offset fell outside two standard deviations from the mean. Finally, saccades greater than 500 ms were removed. In total, 10.7% of trials were excluded from analysis.

An outlier analysis was also conducted for the TOJ reports obtained during the *ceiling* condition in Session 2 and Session 3 to determine if participants were able to successfully perform the desired tasks. The assumption was that since all participants in Session 1 performed significantly above chance in higher SOA trial bins and since previous studies already demonstrated that participants can consciously perceive a temporal difference above ∼65 ms (Chassignolle, Giersch, & Coull, 2021; Stelmach, Campsall, & Herdman, 1997), any participants who performed at or below chance level during the ceiling condition may have misunderstood the task and should be excluded. This was the case in two observers whose data sets were consequently excluded from all analyses.

### Data analysis

Data were analyzed using MATLAB scripts and R studio statistics software (R Core Team, 2021). Trials from Session 1 were analyzed using a psychometric function to determine each observer’s individual just noticeable difference value (JND) value. In the following, we describe the procedure used to compute this value. Psychometric functions were fitted online using the *psignifit* 2.5.6 toolbox for MATLAB, which implements the maximum likelihood method described by Wichmann and Hill (2001). Functions were refitted offline using the QuickPsy library in R studio (see Linares & López-Moliner, 2016, for more information). The data were fitted as a cumulative normal function derived from the linear stimulus scale with a y-axis value of between 0 and 1 (accuracy frequency) and an x-axis showing the SOA intervals. The guess rate was fixed at 0.5, the value typical for 2-alternative forced choice (2AFC) experiments. The 75% threshold score was used as the JND value.

The JND threshold from Session 1 was interpreted as the point at which participants were able to indicate reliably (i.e., number of correct responses are significantly above chance) which of the two stimuli appeared first and which appeared second. This value was then preloaded as the critical condition (*JND* condition) for Session 2.

For Session 2, accuracy (e.g., percentage of correct responses) was chosen as our dependent variable. However, the simultaneous condition for both Sessions 2 and 3 was compared separately with response frequency as the dependent variable. The analysis for and findings from this condition are presented in a separate section (see Simultaneous Condition). First, accuracy percentages were transformed into their equivalent arcsine using the following formula, where *x* is the percentage value and *t* is the transformed score:

$$\begin{array}{c} t=\sin^{-1}\left(\sqrt{\left(x÷100\right)}\right)\; \end{array}$$

This was to ensure a continuous normally distributed data set and to avoid violating this assumption, particularly in the *Ceiling* condition, where we expect that most accuracy values will be close to 100%. Arcsine data were then verified for goodness of fit using the Dodge (Dodge, 2008, pp. 12–14) of normal distribution. For clarity purposes, subsequent figures in Results display percentage-based accuracy, rather than arcsine scores.

Repeated-measures multilevel linear models were conducted in order to account for predictor variables (SOA, Saccadic Stimulus, or Dimension) being constructed from dependent data. This design attempts to simulate participant variability for responses to different predictors by way of introducing random effects in order to simulate error rate (Field, Miles, & Field, 2012). Linear models using the method of maximum likelihood estimation with random effects were created using the nlme package in R (Pinheiro et al., 2017) and compared directly to a baseline model with the predictors absent. From this, a likelihood ratio was computed to determine significance.

Session 3 was first checked for eye fixation as defined in Exclusion Criteria. Reaction times were then compared to Session 2 by simple two-sample *t*-tests. A repeated-measures multilevel linear model was computed looking at the effect of *Saccadic Stimulus* to determine whether there was a difference between saccade- and non-saccade-related trials that performed the same TOJ. As explained above, a separate analysis was conducted for the simultaneous condition (see Simultaneous Condition).

In the following, we describe the factors used in our analyses. Sessions 2 and 3 had separate linear model analyses conducted. Of primary interest was TOJ accuracy, so this was the fixed-effect dependent measure. First, as described in the procedure, the factor SOA had two levels for Sessions 2 and 3: *JND and Ceiling.* Next, the factor *Saccadic Stimulus* was defined by the relationship of the location of specific elements of the probe and spatial goal of the saccade. So, *First* trials would be trials where the arrow pointed at the target that appears first, which is predicted to be further enhanced via the prior entry effect, whereas *Second* trials would refer to trials where the arrow pointed at targets that appeared second and thus interfered with temporal judgments. A further level of *Other* was used for trials where the arrow pointed at neither of the two targets. Therefore, the factor Saccadic Stimulus had three levels: *First*, *Second*, and *Other*. To examine the extent to which attention is spatially distributed, we introduced the additional factor *Directionality* in our analysis. This factor included the following levels: *Horizontal*, *Vertical*, and *Diagonal* trial types. An overview of the different conditions and how they were categorized is shown in Table 1.

## Results

### Session 1

**Figure 2.**
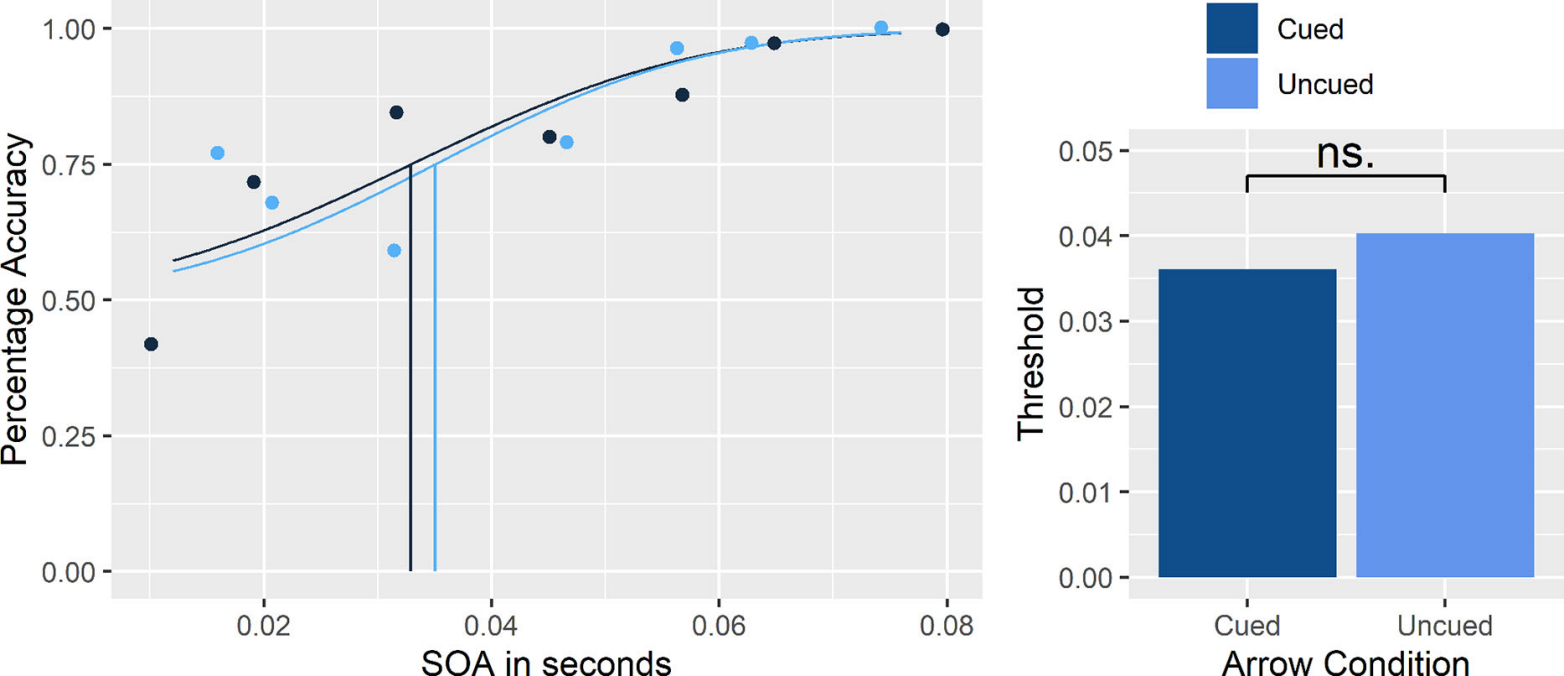
Psychometric function of representative participant accuracy threshold*.* Psychometric function of the cue (dark blue) and no-cue (light blue) conditions plotted as a function of SOA and accuracy. Vertical line represents the *Just Noticeable Difference* threshold for .75 accuracy. The x-axis refers to the different SOAs measured in seconds (on the left) and the two arrow conditions (on the right). The right side of the figure demonstrates the threshold averaged across all participants.

Participants’ TOJ responses, recorded as either correct or incorrect, were plotted using 2AFC psychometric functions. JND thresholds were computed as the SOA value at which, along the psychometric function, a performance of 75% correct judgment score was first achieved (calculated as .75 accuracy). Figure 2 shows a representative participant's results for accuracy for all SOA conditions (binned). The accuracy values (% correct) were summarized across the different response directions (up vs. down/left vs. right) and response types corrected (“which came first” and “which came second”). The right side of the figure shows the overall difference of all participants. Next, *cue* and *no-cue* trial TOJ accuracy was compared to determine if the uninformative arrow cue caused any overall difference in participant response behavior. No significant difference between the two conditions was found (*t*(14) = −0.11, *p* = 0.5), see Figure 2 (right side). This implies that the introduction of the arrow cue did not alter response behavior when compared to trials where the arrow was absent. This means that the accuracy data were similar for *cue* and *no-cue* trials. As this was anticipated, we decided to use data from all trials to compute the relevant, observer-specific JNDs and observer-independent SOA conditions for Session 2. The absence of an overall difference between *cue* and *no-cue* trials does not, however, preclude the possibility that within the class of cue trials, differences between the different types of cue trials might emerge. With that in mind, we examined the differences between the three types of cues (Cue First, Cue Second, Cue Other) and found no significant effects. This indicates that in the absence of a required attentional shift, no benefits were garnered based on the uninformative probe. The threshold JND scores averaged from all Session 1 trials (*M* = 0.395, *SD* = 0.161) were computed and transferred via script, online, to Session 2 for each participant to use as the critical *JND* SOA condition.

### Session 2

After controlling for exclusion criteria, saccadic reaction times (sRTs) were computed as the duration of the time interval beginning with the GO signal and ending with the eye arriving at the endpoint of the saccade (*M* = 0.2, *SD* = 0.08). sRTs in our study, averaged across participants, were within the typical range described for goal-directed saccades (Fischer & Ramsperger, 1984; Gezeck, Fischer, & Timmer, 1997). In over 80% of trials, the saccade started between 100 and 200 ms after the GO signal tone.

**Figure 3.**
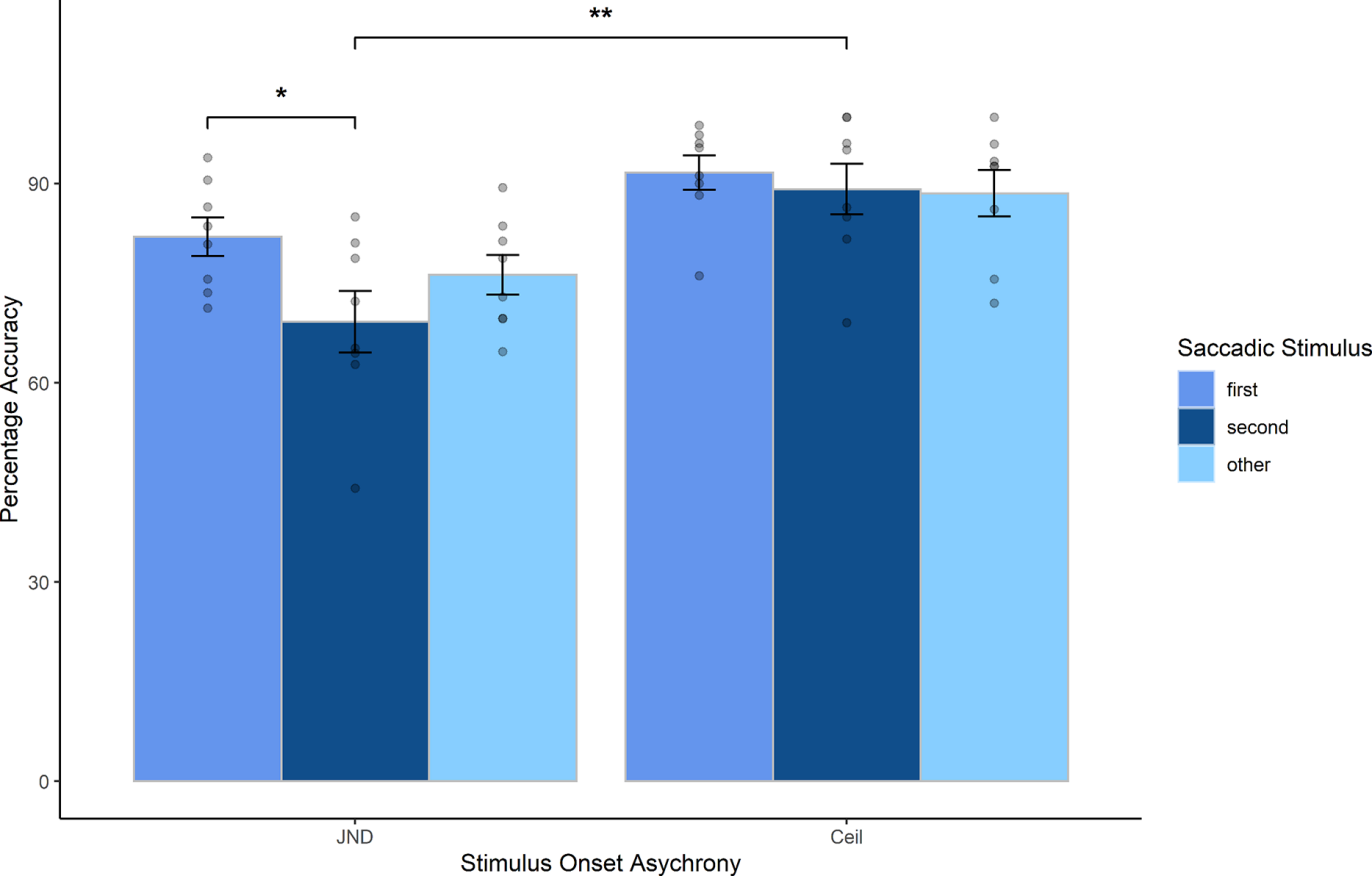
Mean accuracy difference for SOA and Saccadic Stimulus conditions. Mean within-subject accuracy for the SOA condition of Session 2. Conditions are further separated into their Saccadic Stimulus conditions. *First* refers to trials where the cue pointed to the first-to-appear probe, *second* refers to trials where the cue pointed to the second-to-appear probe, and *other* refers to trials where the cue pointed to an irrelevant location. Scatterplots show individual data points. Data were analyzed using arcsine transformed values but are presented here using percentages for clarity.

First, it was important to ascertain that there was a clear distinction between the SOA conditions (*JND* and *Ceiling*). This analysis aimed to examine whether SOA was effective in manipulating participants’ uncertainty. A paired samples *t-*test revealed a significant difference between the means of *JND* (*M* = 75.8) and *Ceiling* (*M* = 89.9), *t*(7) = −4.4374, *p =* 0.003. Next, the critical *JND* condition, where the effect was expected to be strongest, was examined in order to analyze the effects of attentional manipulations. Therefore, a factorial repeated-measures multilevel linear model was constructed for the JND condition with accuracy (transformed arcsine) as the fixed-effects dependent measure, *Saccadic Stimulus* (*First*, *Second*, and *Other*) as fixed-effects independent factors, and participant as a random effect. As a brief reminder, the Saccadic Stimulus conditions refer to which probe target the arrow cue pointed. For example, if it pointed at the first-to-appear stimulus, this is a *First* trial. This analysis allowed us to examine the effects of the cuing manipulation by exploring whether the spatial congruence between saccade endpoint and stimulus location affected the TOJ responses. *Saccadic Stimulus* had a significant effect on accuracy, χ^2^(8) = 8.42, *p* = 0.014 (see Fig. 3). Orthogonal contrasts within the model further revealed that there was a significant difference between accuracy of overall First trials and accuracy of Second trials, *b* = 0.07, *t*(14) = 3.114, *p* = 0.007. Tukey's HSD (honestly significant difference) test, performed post hoc, revealed a significant difference, with *First* (*M* = 82.0) being significantly more accurate than *Second* (*M* = 69.2), *t*(28) = 3.114, *p* = 0.02, but no significant difference between *First* and *Other* (*p* = 0.32) or *Second* and *Other* (*p* = 0.28)*.* Taken together, these results demonstrate that participants were significantly more accurate at judging which target came first/second when performing a saccade toward the probe that appeared first in JND SOA trials. No effects had been anticipated for the *Ceiling* condition, and all responses across the *Saccadic Stimulus* conditions remained close to 90%. Nonetheless, a multilevel linear model was conducted and found no significant difference between the three *Ceiling* conditions, χ^2^(7) = 0.51, *p* = 0.77 (see Figure 3). This demonstrated that participants were following the instructions correctly and served as effective catch trials to rule out random responses or guessing.

Next, directionality of attentional spread was looked at. No direct predictions had been made about the relationship between directionality and attention, but the design presents an opportunity to look at the spatial distribution of attention in more detail. Previous research has demonstrated the effects of hemispheric manipulations on a variety of attentional tasks (see Discussion), and thus an exploratory post hoc analysis was conducted. The *JND* condition was used for this analysis to allow for a clear depiction of how attention could be primed even in the case of objectively sequential probes. Directionality refers to the distribution of the target probes across the different hemifields. So, for example, a horizontal trial would involve probes where one probe is in the right and the other in the left half of the monitor, but they both appear at the same vertical height. Conversely, in vertical trials, probes are dispersed between the upper and lower halves of the monitor, and again, both probes are to be found at the same horizontal distance to the fixation point. In diagonal trials, horizontal and vertical coordinates of the two probes are different.

**Figure 4.**
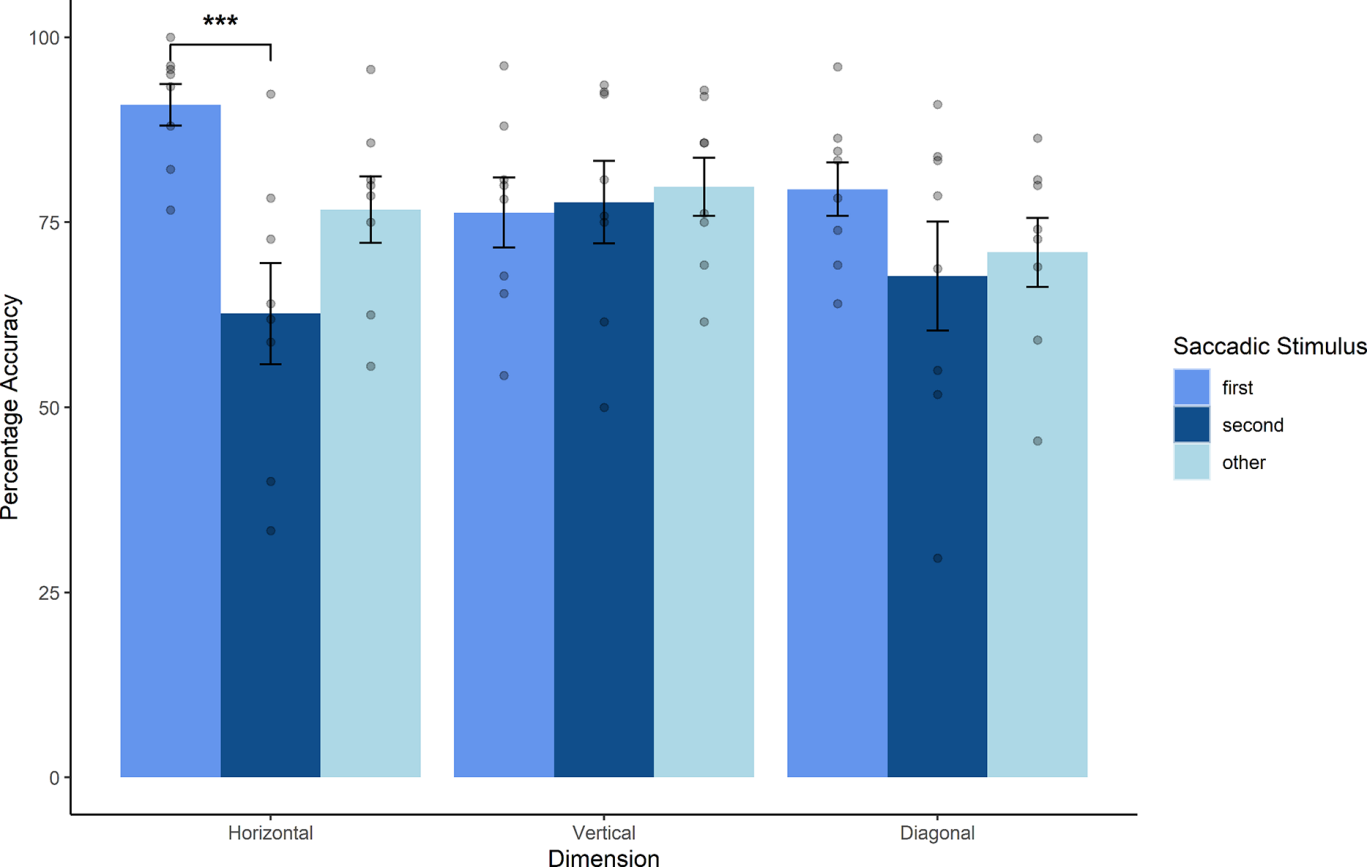
Dimension data comparison. Mean accuracy of within-subjects accuracy for the JND condition of Session 2. This is shown for all three Saccadic Stimulus conditions. The x-axis refers to the different dimensional configurations. Scatterplots show individual data points. The difference between first and second arrow-pointing condition was significant in the horizontal condition.

A factorial repeated-measures multilevel linear model GLM (General Linear Model) was constructed. Accuracy was defined as the dependent variable, and Saccade Stimulus (First, Second, and Other) and Directionality (Horizontal, Vertical, and Diagonal) were the two factors. The Saccade Stimulus had a significant impact on participant accuracy, χ^2^(3) = 8.43, *p* = 0.015. Most important, the Directionality by Saccade Stimulus pointing direction interaction was also significant, χ^2^(5) = 13.78, *p* = 0.008. Orthogonal contrasts revealed that there was a significant accuracy difference between the two Saccade Stimulus conditions (First vs. Second), *b* = −0.17, *t*(14) = 2.53, *p* = 0.02. Post hoc Tukey HSD multiple comparisons demonstrated that Saccade Stimulus First and Second probe trials were significantly different only in the Horizontal dimension (*p* < 0.001). See Figure 4.

Taken together, these results demonstrate that direction did influence participants’ responses, but only when attention was modulated. Diagonal trials were found to be on average more difficult, especially in comparison to horizontal trials. The combination of *Cue First* arrow and *horizontal* trial placement led to the highest accuracy (∼82%), whereas the lowest accuracy trials were found in the *Cue Second Horizontal* trials (∼69%). The interaction between *Saccadic Stimuli* and *Directionality* shows that the presaccadic attentional shift resulted in the most pronounced prior entry effect when probes were separated between the two horizontal hemifields. One interpretation that can be taken away from this is that premotor attention spreads more effectively within one horizontal hemifield but not across the two lateral hemifields, as can be seen in Table 2. The prior entry effect is isolated to trials where the two probes are presented in the horizontal condition (as confirmed by a higher accuracy performance in *Cue First* trials than in *Cue Other*). For trials in the *vertical* as well as *diagonal* conditions, no significant differences between *Saccadic Stimulus* conditions were found.

**Table 2.** Mean accuracy of trials shown for directionality vs. Saccadic Stimulus.

	Directionality		
Saccadic Stimulus	Horizontal	Vertical	Diagonal
Cue First	90.86	76.31	79.46
Cue Second	62.67	77.7	67.72
Cue Other	76.72	79.78	70.93

### Session 3

For Session 3, it was critical to ascertain whether the arrow cue alone—even if that cue did not serve as instruction for a saccade and was noninformative—was influencing attention. Table 3 shows the mean accuracy TOJ scores for the different *Saccadic Stimulus* for both Sessions 2 and 3. Because cue-dependent effects were established in Session 2 in the *JND* condition, the same analysis was conducted here for Session 3. A factorial repeated-measures multilevel linear model was constructed with accuracy (transformed arcsine) as the fixed-effects dependent measure, *Saccadic Stimulus* (*First NS*, *Second NS*, and *Other NS*) as fixed-effects independent factors, and participant as a random effect. No significant effect of *Saccadic Stimulus* was found, χ^2^(7) = 0.18, *p* = 0.91. Consequently, no post hoc comparisons were conducted. It is therefore demonstrated that the cue was no longer influential in facilitating attentional effects when the saccade preparation was no longer required at the critical SOA timing.

**Table 3.** Mean accuracy values of sessions 2 and 3.

	SOA	
Saccadic Stimulus	JND	Ceiling
Session 2		
Cue First	82.0	91.6
Cue Second	69.2	89.2
Cue Other	76.3	88.5
Session 3		
Cue First NS	73.6	93.3
Cue Second NS	71.8	89.9
Cue Other NS	72.0	92.0

### Simultaneous condition

#### Simultaneous session 2

The simultaneous condition for both Sessions 2 and 3 was compared separately with response frequency as the dependent variable. This was because there was no longer an objective accuracy criterion for the participants’ responses. We wanted to test the following hypothesis. Saccades to a given location lead to a shift of attention to this location and consequently to a prior entry effect for probes presented at that location. This can be tested in the following way. First, we divide all trials in saccade-relevant and saccade-irrelevant trials. Saccade-relevant trials are those where one of the probes for the TOJ was presented at a location that was also used as the saccade goal. Saccade-irrelevant trials are trials in which neither of the two probes were at the same location as the saccade goal. Saccade-irrelevant trials were uninformative in this context and thus excluded from the analysis. Now we can look at the TOJ responses in the saccade-relevant trials. On the basis of our hypothesis, we predict that observers will be more likely to select that event or that probe as the first event that appeared at the same location that served as the saccade goal (*saccade-congruent* location; we call the other location the *saccade-incongruent* location). Put differently, we predict that the saccade-congruent probe is selected more frequently than the saccade-incongruent probe. We tested this hypothesis for all three types of spatial classes of trials (see Results, Session 2): horizontal trials, vertical trials, and diagonal trials using a paired-samples *t*-test.

**Figure 5.**
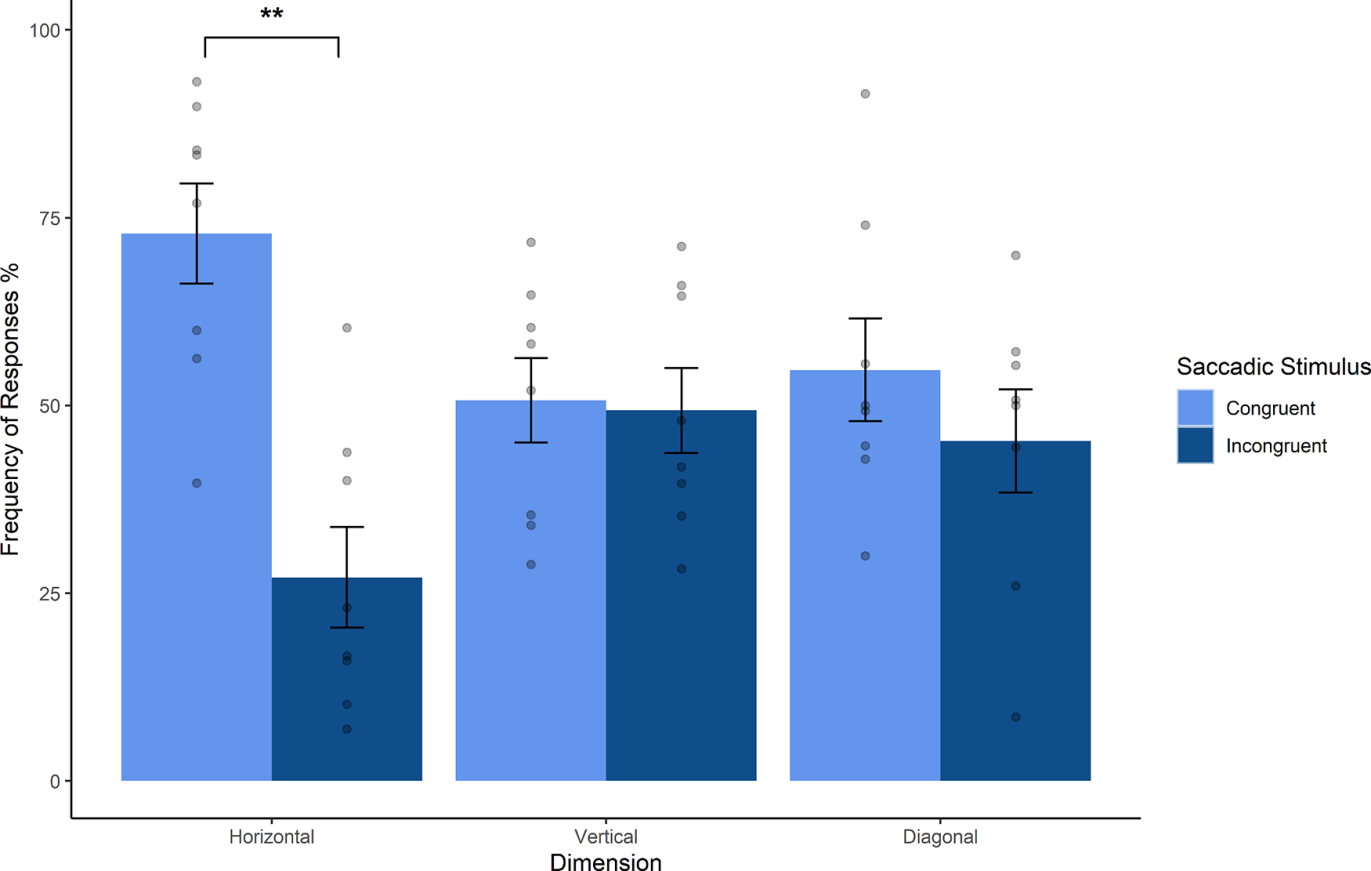
Dimension data of simultaneous condition comparison. Mean within-subject accuracy for the *Simultaneous* condition of Session 2. Saccadic stimuli are now subdivided into congruent and incongruent conditions. The x-axis refers to the different dimensional configurations. Scatterplots show individual data points. The difference between first and second arrow-pointing condition was significant in the horizontal condition.

A higher response frequency at the saccade-congruent location would indicate that participants perceived that location as appearing first and thus had an increased prior entry bias. Trials were no longer subdivided into *First* and *Second* conditions but now measured in total, the number of trials where participants responded in the same direction as their saccade as a percentage of all trials at that location. Paired sample *t-*tests were performed and demonstrated that while across all spatial distributions, there was a slight but not significant trend for a higher frequency of responses to the saccade-congruent location, *t*(7) = 1.83, *p* = 0.11, a significant difference did emerge once we looked at specific spatial configurations. Paired sample *t-*tests revealed that in the horizontal condition, participants responded significantly more often to the saccade-congruent location, *t*(7) = 3.43, *p* < 0.01. But no such significant differences were found for the vertical (*p* = 0.9) and diagonal (*p* = 0.5) conditions; see Figure 5. This analysis demonstrated that participants respond in the direction congruent with their saccade in the horizontal condition and therefore indicates that a prior entry effect exists across the lateral hemifields only, consistent with the directionality analysis conducted for Session 2.

#### Simultaneous session 3

The same analysis was conducted for Session 3 in order to ascertain if participants responded in the direction as the cue in the absence of a saccade (No-Saccade condition, *NS*). A paired samples *t*-test confirmed that participants did not respond more frequently in the cue-congruent *NS* direction than in the cue-incongruent *NS* direction, *t*(7) = −0.6, *p* = 0.56. Therefore, due to this lack of trend for a bias toward the cued location and also because of the smaller number of trials overall, the directionality analysis was not conducted for the data from Session 3.

## Discussion

This study demonstrated robust prior entry effects when participants were required to prepare and execute a saccade to one of two temporally sequential probes. This effect was not observed when participants performed the same task without a saccade, indicating that the motor action (i.e., the saccade) rather than the arrow cue was influencing the attentional shift. As the arrow cue was uninformative, participants gained no strategic benefit by attending to the probed location. On the contrary, as with other AAL designs, it was beneficial to maintain a ubiquitous spread of attention. Since in any given trial, the two probes could appear at any of the placeholder positions presented uniformly across the display, attention should not be restricted to a singular position or configuration. Accordingly, any prior entry effect observed in such a paradigm implies that participants’ spatial bias had been influenced by an automatic shift of attention toward a cued location. As identical trials without saccade produced no such effect, it is evident that this attentional effect is presaccadic in nature and therefore consistent with the PTA. As predicted, trials where participants were instructed to saccade toward the first-to-appear target produced a prior entry effect measured, in this case, as an increase in accuracy in these trials. In contrast, trials instructing saccades to the second-to-appear target produced a prior entry effect in the opposite direction, measured as a decrease in accuracy. By shifting attention to the second target, it is perceived subjectively and, in accordance with the prior entry paradigm (Spence & Parise, 2010), as being presented earlier than it was. Consequently, the interpretation of the temporal gap between first and second target becomes more ambiguous, and the TOJ subsequently becomes more difficult. Finally, trials that instructed participants to saccade to an irrelevant target produced TOJ accuracy comparable to no-saccade trials (∼75%); incidentally, this percentage accuracy fits well with the established threshold ratio defined by Session 1, meaning that when preparing a saccade to an irrelevant stimulus, or else not performing a saccade, accuracy remained close to baseline.

As expected, SOA conditions were influential for determining successful prior entry effects. The *Ceiling* condition did not produce prior entry effects; at this temporal displacement, participants could reliably distinguish the sequential order and therefore served as suitable catch trials to confirm participants were following instructions, whereas the *JND* condition was intended to serve as the condition most sensitive to a biasing of spatial attention. Indeed, robust prior entry effects were observed in the *JND* condition consistent with our expectations. At this SOA, uncertainty was considerably higher, and therefore the perceived temporal displacement is more susceptible to attentional distortions. The prior entry bias is typically subtle, producing effects often below 20 ms for endogenous cuing paradigms (Spence & Parise, 2010), and therefore could be pervasive enough to bias a condition on the threshold of uncertainly while remaining immune to displacements in above-threshold conditions (such as the *Ceiling* condition). Likewise, *simultaneous* trials also produced prior entry effects, primarily in horizontal trials in saccade conditions, with participants’ responses often biased toward perceiving the saccade-congruent location as appearing first. Observing these effects in a simultaneous condition is a convincing indicator that a perceptual bias is present, as participants are at a maximum level of uncertainty due to the two probes appearing concurrently.

One interesting observation was the influence of *Direction* and attention on trial accuracy. It was observed that attentional modulation was influential in the horizontal configuration on presaccadic trials, but the same effects were not observed for the vertical or diagonal conditions. This indicates that presaccadic shifts of attention may be most prominently biased in observations that occur across the left and right of our field of view. In other words, if the two stimulus locations are presented to two opposite horizontal hemifields (e.g., one in the left hemispace, the other in the right hemispace), the effect of preparing a saccade to either one or the other location is particularly pronounced. Specifically, the TOJ accuracy difference between attention on the first versus second stimulus was largest when the two stimuli were presented to the left and right of the observers’ view on the same vertical midline. Effects were somewhat diminished when looking at diagonal trials and had all but vanished when examining purely vertical trials. It could be argued that the moderate albeit less pronounced attentional boost observed for diagonal trials is the result of conflicting modulatory effects of both the horizontal and vertical midline.

Demonstrating that presaccadic attentional effects are spatially influenced may open up further research avenues. Previous studies have looked at horizontal versus vertical visual TOJs but have typically employed display stimuli centered on the vertical or horizontal midline (see Shore et al., 2001) and have not directly explored presaccadic effects. For example, Zampini and colleagues (2003) found, when exploring the influence that spatial distribution has on TOJ discrimination, that multisensory TOJ effects vanished when audio and visual stimuli were presented from the same hemifield. It was argued that the two cerebral hemispheres may contain separate pools of attentional resources and that TOJs could be more accurate if this processing occurred across these pools rather than isolated in one. This hypothesis could explain our results, which seem to indicate that attentional focus is insulated hemispherically. Furthermore, the authors argued that increasing the functional cerebral distance between two probes may also be a factor in minimizing uncertainty. However, the far smaller effect observed in the diagonal condition of our experiment is inconsistent with this cerebral distance hypothesis. Overall, it seems likely that the effects observed for Zampini and colleagues are similar in origin to the findings of our experiments. However, the neurological mechanisms involved in producing this effect are still open to interpretation.

Our study was not the first to examine the role of saccades and how they affect responses in a TOJ paradigm. Stelmach and colleagues (1997) explored the merits of indexing attentional allocation in a TOJ experiment that used prepared saccades to target locations. However, their research focus was not on examining the effects of premotor shifts of attention on TOJ, and therefore their study design was not optimized for exploring those premotor effects. In the following, we describe some of the difficulties of the study by Stelmach and colleagues (1997). First, they had no saccade preparation window and simply instructed participants to perform a saccade as soon as they were cued. While they did test endogenous cueing of the saccade using a verbal instruction, they instructed participants to perform their saccade immediately. Therefore, they did not necessarily measure the same behavioral process when using such a target-triggered saccade as we do with a GO signal–triggered saccade. Indeed, evidence has shown that a preparation time of between 500 and 1,000 ms can help with the programming of a saccade to its target location in endogenous cueing paradigms (Deubel & Schneider, 1996, 2003). Second, their employed control conditions were insufficient to rule out other influences that may have biased their results. For example, in Stelmach et al. (1997), the cue was always informative as there were only two target locations, so the arrow always pointed to a probe location. The absence of neutral trials (i.e., trials where none of the probes appeared at a cued location) might have shaped the attentional-deployment strategy for participants in the study by Stelmach and colleagues (1997). In order to address these issues, our study offers the following additions: First, we tried to avoid the problem of the arrow always being informative by using four placement locations. This allowed us to compare uncued locations and, thereby, rule out top-down cue biasing attentional effects. Second, our study is designed to specifically measure the attentional shifts predicted by the premotor theory of attention (i.e., a shift of attention occurring between the onset of an instructive cue and the onset of the saccade). To maximize the impact of this presaccadic shift, we introduced a delay between cue onset and a GO signal that instructs participants to start their saccade. In addition, our study has a much finer temporal resolution, controls for response effects (response biases) by employing both “which came first?” and “which came second?” response types (see Spence & Parise, 2010), and has the ability to measure anisotropies in the allocation of attention (see preceding paragraph).

### Limitations and future considerations

Due to time constraints, the Session 3 control experiment was not as long or as robust as Session 2. There were half as many trials and the cue window was shorter. It is possible that these limitations may have lowered the power of Session 3 results. It also meant that there were an uneven number of response-type blocks, with participants either having one or two “which-came-first” or “which-came-second” blocks due to the limited number of blocks and counterbalancing. We do not believe that this will have had any meaningful effect on the data, but it is worth repeating that in future designs, the control task could be improved by being the same length as the main session.

## Conclusion

In conclusion, the PTA and, more broadly speaking, all work related to AALs have almost exclusively relied on single-probe stimulus discrimination tasks. To confirm that effects in these paradigms reflect the workings of a task-transcending mechanism of spatial attention, it is important to demonstrate the effectiveness of AAL in other paradigms that use different stimuli and different tasks but can still be seen as tasks measuring the effects of spatial attention. This was accomplished by using a measure that is well established to be a reliable indicator of attentional shifts—namely, the prior entry effect using a TOJ design. The TOJ task allows us to probe attention in multiple places at once in order to ascertain the spatial biases that are caused by attentional manipulations. We successfully established effective prior entry effects through the use of presaccadic preparation and can thus present a novel tool to examine premotor shifts of attention to two locations simultaneously. We further demonstrated a spatial bias in presaccadic attentional shifts that suggests a hemispherically insulated effect.

## Supplementary Material

Supplement 1
