# Supplementary material for: Temporal order judgments and presaccadic shifts of attention: What can prior entry teach us about the premotor theory?
Source: J Vis. 2022 Nov 3;22(12):6. doi: 10.1167/jov.22.12.6 (PMC9645358; doi:10.1167/jov.22.12.6)
Supplement: Supplement 1 [file jovi-22-12-6_s001.pdf]

Supplementary Material

Methods

Apparatus

G-Sync technology along with Variable Refresh Rate (VRR) support allowed for much more fine-grained flip rates between successive screen vertical retraces. The functionality of screen retracing and flip rate are detailed elsewhere in the PsychToolBox documentation. In theory VRR allows for more variable inter-stimulus intervals when combined with the requisite hardware. Therefore, we were able to more accurately tune our Just Noticeable Difference thresholds. Using software developed by PTB (VRRTest script), we were able to test this efficiency of our setup. An average difference between requested frame length and hardware recorded frame rate was *M* = 1.157, *sd* = 0.34 msecs with a median difference of 1.21 msecs, see Figure 1. Furthermore, see Poth et al., (2018) for a more detailed review of VRR technology.

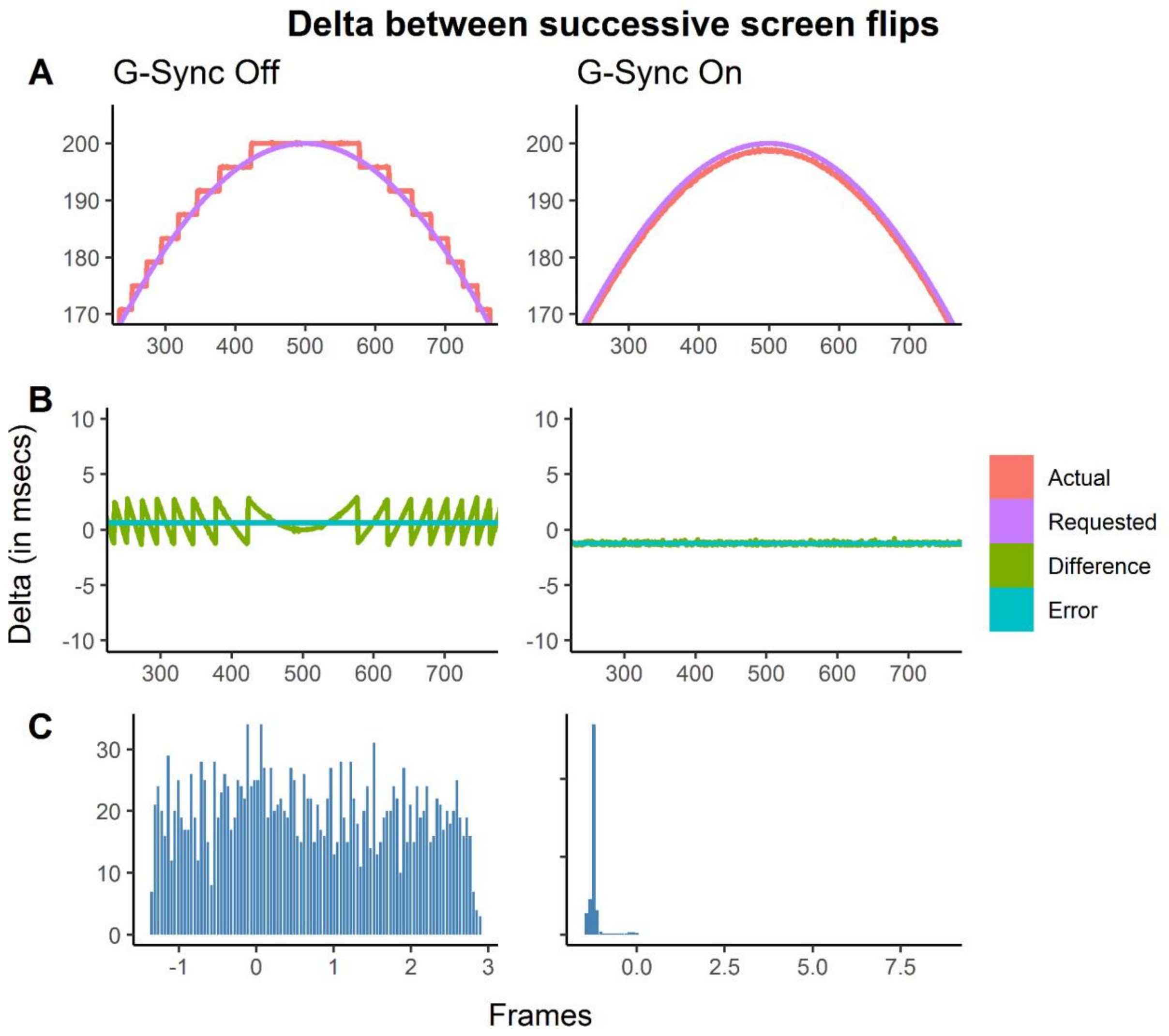

Figure 1 supplementary material. Delta between successive screen flips: Results of adaptive sync mode in PsychToolBox. It submits OpenGL bufferswaps/flips of varying delay between successive flips and then measures and plots how well the hardware can follow the requested timing. Figures on the left side represent results with G-Sync technology switched off, and figures on the right show the results with G-Sync turned on. The X axis for all displays refers to the number of frames. **A** represents the actual measured delay (red) between successive flips against the requested optimal duration (purple). **B** shows the difference (green) and median error (blue). **C** shows a histogram of the difference between requested and actual frame duration in msecs.

Procedure

Participants were required to respond to different combinations of probes in a particular manner. There were three possible probe distributions: horizontal, vertical, and diagonal. See *Figure 2* below for more specific details.

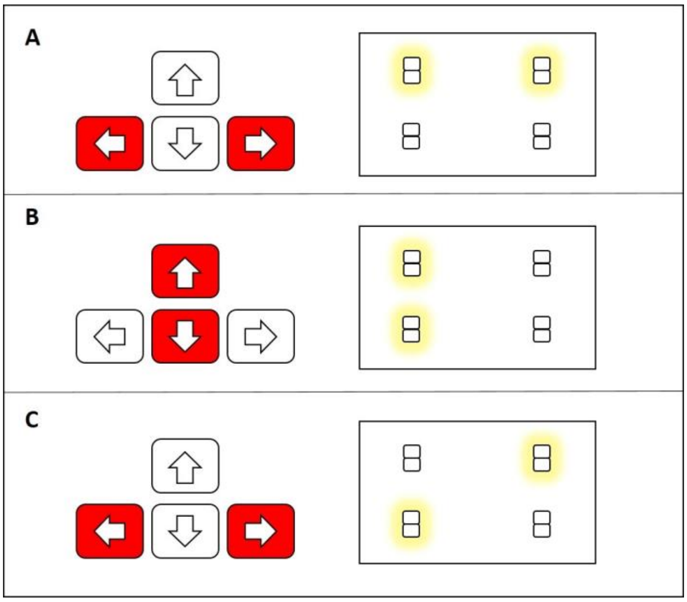

Figure 2 supplementary material. An image detailing the way that key mapping and directionality worked in the study. **A** demonstrates a typical horizontal trial, as can be seen a horizontal trial was when the participant perceived the two probes crossing the vertical meridian but not the horizontal. This required participants to respond with left and right arrow keys. **B** shows a typical vertical trial. Here the participant would respond with up and down arrow keys whenever the two probes crossed the horizontal meridian but not the vertial. Finally **C**, this demonstrates the diagonal condition. This occurred when participants were presented with probes that crossed both the horizontal and vertical meridian and required a key response using left and right arrow keys. Participants were simply taught that any time the two probes crossed the vertical meridian (appeared left and right of fixation) they would respond with the left and right arrow keys. Therefore this would require them to use left and right for both horizontal and diagonal, and up and down for vertical. All participants learned this response criteria quickly and no problems were observed during testing. Incorrect key responses were diasabled to help consolidate this response criteria.

Data Analysis

Binning Data

Due to the large amount of SOAs, online data fitting of Session 1 was binned according to the total number of trials, and derived by the following formula dividing the range of SOAs by the square root (rounded up) of number of trials (*n*) in order to determine the number of bins rounded to the nearest whole number (*t*):

$$\frac{(min^{SOA} - max^{SOA})}{\lceil \sqrt{n} \rceil} \approx t(bins) \tag{1}$$

Reaction Times

For session 1, manual reaction times (RT) were recorded as the time difference between the onset of the first target and the time of the button press response. This onset was used to ensure consistency between reaction times despite varying SOAs. As the information required for making a choice is technically already available after the onset of one target. It is possible that reaction time data can be used to estimate certainty even in the absence of speeded responses in task requirements (Weiß & Scharlau, 2011). Therefore, a simple linear regression was performed to see if there was any influence of SOAs on reaction time. No significant correlation (r^2 = < 0.01, p = 0.49) was found

References

Poth, C. H., Foerster, R. M., Behler, C., Schwanecke, U., Schneider, W. X., & Botsch, M. (2018). Ultrahigh temporal resolution of visual presentation using gaming monitors and G-Sync. *Behavior research methods, 50*(1), 26-38.

Weiß, K., & Scharlau, I. (2011). Simultaneity and temporal order perception: Different sides of the same coin? Evidence from a visual prior-entry study. *Quarterly Journal of Experimental Psychology, 64*(2), 394-416.
